# Mental Health Services Use Predicted by Number of Mental Health Problems and Gender in a Total Population Study

**DOI:** 10.1155/2013/247283

**Published:** 2013-04-04

**Authors:** Maj-Britt Posserud, Astri J. Lundervold

**Affiliations:** ^1^Department of Child and Adolescent Psychiatry, Haukeland University Hospital, 5021 Bergen, Norway; ^2^Regional Centre for Child and Youth Mental Health and Child Welfare, Uni Health, Uni Research, P.O. Box 7810, 5020 Bergen, Norway; ^3^Department of Biological and Medical Psychology, University of Bergen, 5020 Bergen, Norway; ^4^K. G. Jebsen Center for Research on Neuropsychiatric Disorders, 5020 Bergen, Norway

## Abstract

We examined the relationship between service use and the number of problem areas as reported by parents and teachers on questionnaires among children aged 7–9 years old in the Bergen Child Study, a total population study including more than 9000 children. A problem area was counted as present if the child scored above the 95th percentile on parent and/or teacher questionnaire. A total number of 13 problem areas were included. Odd ratios (ORs) for contact with child and adolescent mental health services (CAMH), school psychology services (SPS), health visiting nurse/physician, and school support were calculated with gender as covariate. The number of symptom areas was highly predictive of service use, showing a dose-response relationship for all services. Children scoring on ≥4 problem areas had a more than hundredfold risk of being in contact with CAMH services compared to children without problems. The mean number of problem areas for children in CAMH and SPS was 6.1 and 4.4 respectively, strongly supporting the ESSENCE model predicting multisymptomatology in children in specialized services. Even after controlling for number of problem areas, boys were twice as likely as girls to be in contact with CAMH, replicating previous findings of female gender being a strong barrier to mental health services.

## 1. Introduction

Already in 1979, when elaborating on taxonomy and classification, in view of the coming DSM-III manual, Woods declared that “Substantially supplanting this nineteenth century definition of disease as structural lesion or abnormality is the notion of disease as construct rather than material that exists in the ostensive finger-pointing sense. Diseases of all sorts are constructs that are found useful at different points in time for organizing subject matter” [1]. From hindsight, he might have been a bit too optimistic, as we still in the 21st century tend to overlook this fundamental dogma.

Both in clinical and population studies, psychiatric comorbidity is generally prevalent [2–5] and underresearched, especially regarding treatment, as many trials exclude individuals with certain kinds of comorbidity [6, 7]. Gillberg argued that the very concept of comorbidity prevents clinicians and researchers from providing our patients with the care they need [8]. Comorbidity implies that something exists separated from something else and that there are clear boundaries between one morbus and another morbus. To cite Woods once again, the problem with the word “comorbidity” is that “the data that we see are to an appreciable extent determined by the categories that we apply to them” [1]. Thus the very word comorbidity leads us to believe that the two “comorbid” disorders exist as separate, distinct entities. The symptoms described within the diagnostic criteria for autism and attention deficit hyperactivity disorder (ADHD) are a fragrant example. According to the diagnostic criteria, these disorders are mutually exclusive [9], although recent research (ignoring the diagnostic criteria) points to tight links both genetically and clinically between the two disorders [10–12]. If this is not taken into consideration by clinicians, valuable time and effort may have been wasted and the needs of children neglected. This example demonstrates the dire consequences of believing that diagnoses exist in the absolute sense and in one* exact form* and that diagnostic manuals are idealized but faithful representations of reality.

The large National Comorbidity Surveys carried out in 1990–1992 and 2001–2003 in the US both highlight the high comorbidity rates found in psychiatric disorders and the strong relationship between the presence of comorbidity and severity of the illness [2, 3, 13]. Both found that although half of the entire population was likely to experience at least one psychiatric disorder in their lifetime, only 21% of life-time psychiatric disorders occurred in people who reported only one life-time disorder. In the 1990–1992 study, a sixth part of the population had three or more life-time psychiatric disorders, and nine out of 10 *severe* psychiatric disorders occurred in this group [2]. In spite of their obvious problems, only one-third of them had received any professional treatment in the last year. The same pattern emerged in the 2001–2003 study, where 7% of the population was characterized by having high rates of comorbid psychiatric disorders accounting for 44% of the serious cases [13].

Genetic work including both genome-wide scans, twin studies, and specific genetic deletions/mutations have shown a large overlap between both bipolar disorder, schizophrenia, ADHD, learning disabilities, and autism, indicating that the same genes are involved in these disorders [14–17], but perhaps through varying mechanisms. It should not come as a surprise then that the symptomatology itself may be largely overlapping, and that “specific” disorders perhaps ultimately only exist in an idealized stereotype form. 

In 2010, Gillberg proposed an alternative concept: Early Symptomatic Syndromes Eliciting Neurodevelopmental Examinations (ESSENCE) [8]. Although parents and children usually present with one major concern, the concept calls for us as professionals to know that a difficulty seldom appears on its own. Gillberg claims that the clinical practice of only addressing the presenting problem may be of limited use if the child is struggling in various domains, and that the presenting concern should be viewed more as a warning signal, a cue for the professional to look across domains for more problems. This broader concept may have a cost for the child and his/her family, and the clinician as well as for the society. It is therefore important to investigate the evidence for ESSENCE, as well as the practical and economic consequences of working according to this model rather than following the traditional model of narrowly defined disorders. At the individual clinical level, a narrow approach may be easier to handle in the short-term, but the broader approach may be more beneficial in the long-term and at a societal level. Both approaches may be efficient, depending on where in the school/health services you meet a child. According to Gillberg and the ESSENCE model, we should expect that the children with multiple problems are primarily found among those referred to specialist services [8]. 

Several studies have demonstrated an unequal access to services for girls, where girls are less frequently brought to services, receive their diagnosis later in life, and have unmet needs [5, 18–20]. The reasons for this bias are not well understood, but several studies using case vignettes demonstrate that both parents and teachers seem to rate interventions as less useful for problems exhibited by girls [21, 22]. This prejudice seems to exist also within the health services. One study showed that girls with inattentive type of ADHD, although as likely to be in contact with services as boys, received treatment for their comorbid internalizing disorder rather than for their ADHD [23], and further other studies have shown the reduced likelihood for ADHD diagnosis and treatment for girls, even after adjusting for ADHD symptom severity [20, 24]. Several studies have questioned the frequently cited disruptive behavior hypothesis that boys are more likely to be referred because they show more disruptive behavior, finding that, in vignettes, girls with disruptive behavior were *more* likely to be referred, whereas there was no difference for boys [21]. This study also found parents and teachers beliefs about the efficacy of ADHD treatment to be the mediator explaining the gender bias in seeking health services, and the strongest effect was due to rating teaching assistance as more effective for boys than for girls [21]. Derks et al. (2007) found that although clinically girls and boys with ADHD had the same symptomatology and comorbidity, teacher ratings of boys with ADHD were likely to be higher on inattentive symptoms and aggressive behavior, and the authors speculated that this might contribute to the lower treatment rate for girls [19].

Most of the studies on comorbidity have included clinical samples and are therefore probably affected by participation bias, characteristics of the clinic performing the study, and assessment methods. Population-based studies are thus called for to characterize children at various levels of health services. To that end, the present study examined the rate of reported multiple problems in children at various levels of health/school services in a large total population sample of children the 7–9 years of age. We ask whether, children referred to specialist services show symptom profiles mainly indicating specific symptomatology or a broad range of problems. We further ask whether there is a relationship between the level of service use of the child and the number of problem areas reported, and whether sex impacts on contact with health/educational services after controlling for number of problem areas.

## 2. Materials and Methods

The first wave of the longitudinal Bergen Child Study (BCS) assessed a broad range of mental health problems in a total population of school children aged 7–9 years old (*N* = 9430) through teacher and parent questionnaires [25, 26]. The teacher questionnaires (*N* = 9152) covered 97% of the population, whereas the parental questionnaires (*N* = 6295) covered 67% of the same sample. In the present study, “unidentified children” were defined as children with only (anonymous) teacher reports and no parental report and identifying consent (*N* = 2857), whereas children with parental questionnaire were labeled as “identified children” (*N* = 6295). The main instruments of the questionnaire were the Strength and Difficulties Questionnaire (SDQ), the Autism Spectrum Screening Questionnaire (ASSQ), and the DSM-IV criteria for ADHD and ODD. In addition, there were 5 items targeting obsessive compulsive problems (OCD), 5 items targeting tics, and 5 items targeting language difficulties. All items were scored on a three-point Likert scale (not true, somewhat true, definitely true). There were slight differences between the informants' questionnaires in that parental questionnaires included 5 items on eating disorders and one item targeting sleep problems, while the teacher version included two items on hypoactivity and one item asking for selective mutism. Both informants were asked whether the child, to their knowledge, had been referred to child and adolescent mental health services (CAMH), to school psychology services (SPS), or a health visiting nurse/physician for any of the problems reported in the questionnaire. Response options were “yes,” “no,” or “I don't know.” In the present study, a child was defined as referred to a service if either the parent and/or the teacher reported “yes” regarding that service. 

The problem areas included were the emotional and the peer problem scale of the SDQ, attention, hyperactivity, and oppositional behavior from the DSM-IV criteria, tics, obsessive compulsive disorder (OCD), language difficulties (LD), eating problems (parent only), sleeping problems (parent only), selective mutism (teacher only), and hypoactivity (teacher only) (Table 1). A problem area was defined as present if parent and/or teacher reported a score above the 95th percentile of the whole population sample, except for selective mutism and sleeping problems, where a report of partly true or definitely true was counted as problem present. This procedure corresponded to the 91th percentile for sleeping problems and the 96th percentile for selective mutism. For eating problems, a score of one corresponded to the 96th percentile. For teacher defined OCD, a score ≥2 was used, corresponding to the 96th percentile, as a score of one effectively resulted in a cutoff at the 89th percentile (see Table 1). Each problem area was only counted once (i.e., not twice if reported by both informants). 

### 2.1. Statistical Analyses

Descriptive statistics were used to calculate the frequency of children within each of the problem areas. Logistical regressions analyses were computed separately for identified (teacher and parent reports) and unidentified (teacher reports) children. The number of problem areas, categorically defined, and gender were used as predictor variables and contact with services as outcome variable.

## 3. Results

### 3.1. Characteristics of Children in Contact with Services

Tables 2 to 4 describe the overall number of children and percentages referred to child and adolescent mental health services (CAMH), school psychology services (SPS), nurse/physician, or having any kind of school support.  Table 2 reports results for children where both parental and teacher information is available, whereas Table 3 reports results for unidentified children with only teacher information. As can be seen, many children had some kind of support in school. This service includes all the commonly used interventions in schools, for example, extra reading assistance and increased supervision during meals; still the majority of these children (66%) scored above the 95th percentiles on 2 or more areas. As expected, children in contact with CAMH services were reported to have very high symptom levels, with 73% of children scoring above the 95th on at least four problem areas, with six areas as both mean and median number.

As many problem areas were included, the number of problem areas in children without service contacts were included for comparison. In this group, very few scored high on any area, and only 3.7% scored above the 95th percentile on ≥4 areas (Table 2).

Having anonymous teacher questionnaires for the 30% children without parental consent (unidentified children) in the BCS provided us with the possibility of examining the effect of nonresponse. We compared problem areas in relation to service use using only teacher information in the identified group as well (Table 4), to enable comparison between the identified sample and the unidentified sample. As expected, in the identified group, the number of children reported to be in contact with health services and the number of problem areas they suffer from are lower when only teacher information is used. Unidentified children have higher problem levels and higher rates of referral to services when compared to identified children when based on teacher reports only. 

### 3.2. Predicting Contact with Services

Table 5 shows the odds ratios (OR) for being in contact with child and adolescent mental health services (CAMH), school psychology services (SPS), and health visiting nurse/physician and for having any kind of support in school according to number of problem areas. The relationship between service contact and number of problem areas was very strong for all services, following a dose-response relationship. Having four or more problem areas increased the OR of being in contact with CAMH by 147 times (identified children). 

With the exception of contact with health visiting nurse/physician among unidentified children, male gender was a significant predictor for all service contact. Even when controlling for number of symptoms, boys were 1.8 times more likely to be in contact with CAMH services for identified children (OR 1.8, 95% CI 1.2–2.6) and more than twice as likely in the group of unidentified children with OR 2.4 (95% CI 1.2–4.9).

## 4. Discussion

The symptom load in children in contact with specialized services (CAMH) was high, with children scoring above the 95th percentile on six different areas as a mean. Primary education services, such as any kind of assistance in school, targeted a larger percentage of the population, and children in touch with these services also had a lower symptom load. This supports the notion that children who are referred to specialist services should be assessed broadly, as they are likely to suffer from symptoms from more than one specific problem area. It also supports the idea that this is true mainly for specialized services, as many children at lower levels of services actually do suffer from problems from one or two domains only.

To assess the relative risk of being in contact with various services, logistic regression analyses were performed with number of problem areas as predictor. Gender was entered as well and turned out to be a significant predictor for both CAMH and SPS also after having controlled for symptoms, with boys much more likely to have been in contact with these services than girls. This pattern was even stronger among unidentified children, maybe due to the fact that they only had teachers as informants. Publications from BCS have previously shown that teachers seem to have a stronger gender bias than parents in their ratings of the children [26, 27], and Derks et al. (2007) also found teacher scores to be lower for girls than for boys [19]. The present analyses indicate that the gender bias is even more far reaching, in that even when the symptoms have been consciously registered by the teacher, contact with services is less likely to be available for girls. Kopp et al. (2010) found that girls, in spite of having very disabling symptoms and high levels of comorbid problems, were brought late to services and had a much higher than expected age at diagnosis of ASD and ADHD [5]. The results are all the more alarming as we used the 95th percentile of the total population, whereas gender specific norms are lower for girls for most problem areas in this age group (http://www.sdqinfo.com/UKNorm.html) [28]. This means that compared to their own kind, they are likely to be even more deviant than the boys referred to same specialist services. Ohan and Visser (2009) found the same pattern. The gender bias in their study was driven by the low chances of referral of girls with only ADHD; whereas boys with ADHD only were likely to be referred, girls needed the additional presence of disruptive behavior to be referred [21]. In a population-based study, Sawyer et al. (2004) also found comorbid conditions predictive of service use in ADHD, but only for girls [29]. In two American studies, parents of girls with ADHD reported higher stigma-related barriers to seek help than parents of boys [24] and girls were less likely to receive services in spite of scoring higher on ADHD symptoms [20]. In other words, girls are required to be more impaired before referral is likely. This bias was true for both parents and teachers alike, and the reason for this difference was explained by parental and teacher assumptions that treatment, particularly teaching assistance, would be more efficacious/needy for boys [21]. 

A minor note concerns the difference between identified and unidentified children. We know from previous studies on the BCS sample that unidentified children have higher symptom scores on all areas [25–27, 30]. Still, they are less in contact with services and the children in contact with services demonstrate lower amount of problem areas, if compared with all the available information from both parents and teacher questionnaires. However, when comparing identified and unidentified children on teacher reports only, it is clear that the difference is explained by the lack of parental reports on these children, not due to their having less problems. The teacher is less likely to be informed about the child's health seeking behavior, and we see that the difference in percentage in contact with school psychology services (which the teachers are generally informed about) is less pronounced. Comparing the findings based on one informant versus two informants in the identified children, we see that the number of problem areas is drastically lower, indicating the importance of including several informants, as one informant may not have access to the entire range of behaviours and difficulties the child is exhibiting.

The present study did not investigate the differential contribution of specific symptoms to the service contact. The aim of the study was rather to investigate whether children in contact with specialist services suffer from many different symptom areas rather than from one or two specific problems. A total of 13 symptom areas was included for identified children, and one might argue that, having so many symptom areas, chances are high for any child to score above the 95th percentile. However, of children not referred to any services, only 15.2% had problems on more than one symptom area, and only 3.7% scored above the cutoff on three symptom areas. For children in contact with CAMH more than 90% had at least 2 problem areas, and 73% scored above the 95th percentile on four or more problem areas. The symptom areas covered a wide range of problems, but many common problems among children referred to CAMH, were still not included, such as enuresis, clumsiness, and conduct disorders. It is therefore not unlikely that the present study actually underestimates the width of problems children in CAMH services suffer from. 

When it comes to the gender bias, the disruptive behavior hypothesis has been put forward as explaining why girls are less likely to be referred to school and health services than boys, indicating that we should have analyzed whether differences in specific problem areas contributed to the gender bias. However, several studies have found parental and teachers beliefs about treatment efficacy for girls and boys to be central to explaining the referral bias to CAMH rather than symptoms within the child [20–22, 24], and this bias is further enhanced by the health services, in being less likely to diagnose ADHD and provide adequate treatment for girls [20, 23, 24]. Furthermore, only one problem area targeted the internalizing domain (SDQ-emotional problems) (stereo) typically associated with female symptomatology, making it rather unlikely that this alone would explain the gender differences.

One limitation is only having access to questionnaire ratings. The questions regarding contact with services did not specify when the contact was and the nature of the contact. The main strength is the total population sample included, with teacher questionnaires covering 97% of the population, eliminating the effects of selection bias. Thus, we trust the main conclusion of the study; that as a rule, children in contact with specialized educational and health services are reported to have symptoms suggesting a wide range of mental health problems. These services need to adopt a broad assessment approach in order to adequately meet the needs of these children. Even though one area may cause the referral and be the main problem at one point, the child and its family are unlikely to benefit from specific interventions targeting that single problem if several others occur in conjunction. 

## 5. Conclusion

The ESSENCE model was supported in the present study of a total population of children 7–9 years of age, as almost all children in contact with CAMH suffered from many problems rather than single and specific problems as measured by parent and teacher questionnaires. High scores within several areas were also highly predictive of being in contact with specialized services. A sad finding was that girls were much less likely to be in touch with services, even after controlling for number of symptoms. This indicates that not only are girls struggling unseen, but even worse they are struggling unaided even when being seen. 

## Figures and Tables

**Table 1:** Problem areas and actual percentages of children scoring above the 95th percentile on parental and teacher questionnaires.

Problem area	Parent questionnaire	Teacher questionnaire	Either informant*
SDQ emotion	5.9%	5.7%	9.7%
SDQ peer problems	7.3%	4.1%	8.9%
Hyperactivity	6.4%	5.8%	9.2%
Inattention	6.5%	6.0%	9.1%
Oppositional behaviour	7.0%	5.3%	8.4%
ASSQ	5.9%	5.2%	8.4%
Language problems	6.4%	6.0%	8.6%
Tics	9.8%	6.4%	13.5%
Obsessions/compulsions	10.2%	3.5%	12.5%
Hypoactivity	—	9.3%	7.7%*
Selective mutism	—	4.1%	4.0%*
Eating problems	4.7%	—	4.7%
Sleep problems	8.5%	—	8.5%

*This column only represents identified children with questionnaires from both informants (*N* = 6295), whereas the teacher questionnaire column represent the total population (*N* = 9152), which is the cause of differing percentages of hypoactivity and selective mutism in these two columns.

**Table 2:** Identified children, percent of referred children with ≥2 and ≥4 problem areas according to parent and/or teacher, mean and median number of problem areas above 95th percentile (*N* = 6295).

Service	Number of children	% Children with ≥2 problems	% Children with ≥4 problems	Mean number of problems	Median number of problems
CAMH	*N* = 153	91%	73%	6.1	6
SPS	*N* = 533	79%	54%	4.4	4
Physician/nurse	*N* = 760	69%	43%	3.7	3
School support	*N* = 829	66%	40%	3.5	3
No services	*N* = 4945	15.2%	3.7%	0.7	0

**Table 3:** Unidentified children, percent of referred children scoring above 95th percentile ≥2 and ≥4 problem areas, mean and median number of problem areas above 95th percentile. Information based on teacher information only (lacking sleep and eating problems) (*N* = 2857).

Service	Number of children	% Children with ≥2 problems	% Children with ≥4 problems	Mean number of problems	Median number of problems
CAMH	*N* = 64	80%	59%	4.4	4
SPS	*N* = 267	72%	49%	3.6	3
Physician/nurse	*N* = 247	66%	38%	3.1	3
School support	*N* = 528	53%	31%	2.5	2
No services	*N* = 2223	8.6%	2.2%	0.4	0

**Table 4:** Identified children, percent of referred children above 95th percentile on ≥2 and ≥4 problem areas, mean and median number of problem areas above 95th percentile. Based on teacher information only (lacking sleep and eating problems) (*N* = 6295).

Service	Number of children	% Children with ≥2 problems	% Children with ≥4 problems	Mean number of problems	Median number of problems
CAMH	*N* = 101	81%	62%	4.4	5
SPS	*N* = 398	64%	38%	3.1	2.5*
Physician/nurse	*N* = 327	56%	36%	2.8	2
School support	*N* = 690	46%	23%	2.1	1
No services	*N* = 5420	5.8%	1.0%	0.3	0

*Out of 398 children, exactly 199 had ≤2 symptoms and 199 had ≥3 symptoms therefore the median is exactly between 2 and 3 symptoms.

**Table 5:** Logistic regression analyses showing odds ratios (OR) for services with number of symptoms and gender as predictors, for identified and unidentified children separately.

Predictor	Identified children			Unidentified children		
	Not ref. *N*	Ref. *N*	OR (95% CI)	Not ref. *N*	Ref. *N*	OR (95% CI)
	Child and adolescent mental health					

0 symptoms	3592	5	1 (ref)	1885	9	1 (ref)
1 symptom	1180	10	6.0 (2.0–17.5)	402	3	**1.5 (0.4–5.4) n.s.**
2 symptoms	516	12	15.6 (5.5–44.5)	199	6	5.4 (1.9–15.5)
3 symptoms	281	14	32.6 (11.6–91.2)	103	8	14.3 (5.4–38.2)
≥4 symptoms	494	112	147.2 (59.7–363.0)	199	38	30.0 (14.1–64.2)
Girls	3117	42	1 (ref)	1291	10	1 (ref)
Boys	3023	115	1.8 (1.2–2.6)*	1435	52	2.4 (1.2–4.9)**

	School psychology service					

0 symptoms	3537	61	1 (ref)	1856	38	1 (ref)
1 symptom	1132	59	3.0 (2.0–4.2)	372	33	4.0 (2.5–6.6)
2 symptoms	466	62	7.1 (4.9–10.3)	172	33	8.8 (5.4–14.6)
3 symptoms	230	65	14.8 (10.1–21.5)	83	28	13.9 (8.0–24.3)
≥4 symptoms	322	286	47.2 (34.9–63.8)	102	135	57.4 (37.7–87.6)
Girls	3010	147	1 (ref)	1237	64	1 (ref.)
Boys	2745	393	2.2 (1.8–2.7)	1293	194	1.6 (1.1–2.2)*

	Health visiting nurse/physician					

0 symptoms	3484	114	1 (ref)	1851	43	1 (ref)
1 symptom	1056	134	3.9 (3.0–5.0)	368	37	4.3 (2.8–6.8)
2 symptoms	423	105	7.4 (5.6–9.9)	170	35	8.8 (5.5–14.2)
3 symptoms	205	90	13.0 (9.5–17.8)	78	33	18.2 (11.0–30.2)
≥4 symptoms	289	317	32.4 (25.3–41.5)	138	99	30.9 (20.8–46.0)
Girls	2858	299	1 (ref)	1229	72	1 (ref)
Boys	2664	474	1.2 (1.0–1.4)**	1318	169	**1.3 (0.9–1.7) n.s.**

	School support					

0 symptoms	3438	159	1 (ref)	1749	145	1 (ref)
1 symptom	1059	131	2.6 (2.1–3.3)	310	95	3.6 (2.7–4.7)
2 symptoms	410	118	5.9 (4.5–7.6)	135	70	5.9 (4.2–8.3)
3 symptoms	205	90	8.8 (6.5–11.8)	61	50	8.6 (5.6–13.1)
≥4 symptoms	275	331	24.0 (19.2–30.2)	69	168	26.2 (18.7–36.6)
Girls	2885	272	1 (ref)	1142	159	1 (ref)
Boys	2569	569	1.8 (1.5–2.2)	1133	354	1.5 (1.2–1.9)

*P < 0.01, **P < 0.05; nonsignificant values are marked with n.s. and shown in bold. All other values are statistically significant at *P* < 0.001.
